# Sex and gender differences in the management of chronic kidney disease and hypertension

**DOI:** 10.1038/s41371-023-00843-9

**Published:** 2023-06-27

**Authors:** Kaitlin J. Mayne, Michael K. Sullivan, Jennifer S. Lees

**Affiliations:** 1 School of Cardiovascular and Metabolic Health, College of Medical and Veterinary Life Sciences, University of Glasgow, Glasgow, UK; 2 Medical Research Council Population Health Research Unit, Clinical Trial Service Unit and Epidemiological Studies Unit (CTSU), Nuffield Department of Population Health, University of Oxford, Oxford, UK

**Keywords:** Risk factors, Prognosis

## Introduction

Hypertension is both a precursor to and a sequela of chronic kidney disease (CKD). Hypertension is increasingly prevalent as kidney function declines and occurs in between 60 and 90% of individuals with CKD [1]. Optimisation of blood pressure control reduces cardiovascular risk in CKD and may slow kidney disease progression [1] thus blood pressure control is a critical component of CKD management.

**Fig. 1:**
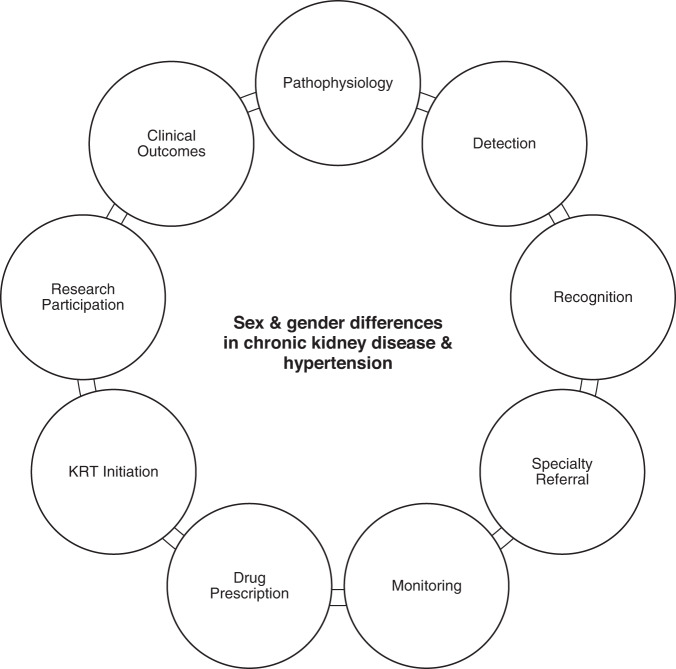
Sources of sex and gender differences in chronic kidney disease & hypertension. Detection = Appropriate biochemical testing: screening of at-risk individuals with hypertension and/or diabetes. Recognition = Diagnosis of CKD if biochemical indicators of disease are recorded. KRT Initiation = Kidney replacement therapy initiation: decision-making regarding modality or conservative care as well as timing of dialysis initiation with respect to kidney function.

In contrast to decreasing trends observed over time for other sequelae of hypertension (such as stroke and ischaemic heart disease), CKD is a growing problem: age-standardised mortality rates in CKD remain stubbornly high [2]; and by 2040, CKD is projected to be the fifth leading cause of death worldwide [3]. Sex and gender have key impacts on many aspects of CKD - including prevalence and patterns of disease, treatment decisions and clinical outcomes (Fig. 1) [2, 4, 5]. Though often incorrectly used interchangeably, sex and gender must be understood as different concepts. Sex is a biologically-determined characteristic reflecting physical differences whilst gender is self-ascribed and reflects socio-cultural factors.

The pathophysiology of CKD appears to differ by sex. Accepting limitations relating to sampling bias and the markers used to estimate kidney function, CKD is reported to be more common in women, yet is thought to progress more slowly compared with men [5, 6]. Perhaps as a consequence, both kidney replacement therapy [7] and age-standardised CKD-related mortality are markedly higher in men [2]: the *Global Burden of Disease Study* (spanning over 20 years) quantified this increased CKD mortality risk at around 30% [2]. Sex differences were broadly consistent across 50 countries, which may suggest biological sex is a greater contributor to CKD-related mortality than socio-cultural gender-related factors.

Hormones are likely to influence sex differences in CKD–whether due to beneficial effects of oestrogen or deleterious effects of testosterone [8]. Though hormones may be particularly influential during reproductive years, the *Global Burden of Disease Study* found that sex differences in CKD-related mortality were remarkably consistent from ages 20 to 90 years [2]. Regardless, sex certainly complicates the prescription of disease-modifying treatment for CKD and hypertension, such as the use of renin-angiotensin system (RAS) inhibitors in women of child-bearing potential. Furthermore, differential treatment of kidney disease by sex and gender is also apparent in the treatment of kidney failure using kidney replacement therapy. Women are more likely to donate a kidney as a living donor [9] but less likely to receive a transplant or undergo dialysis [7]. This reduced use of kidney replacement therapy in women compared with men [7] likely reflects both sex and gender differences. Sex differences might reflect biology of disease progression and severity and in women; pregnancy-induced sensitisation to potential kidney donors reduces access to transplantation [10]. While sex differences are patient-specific, gender differences may be both patient- and clinician-driven: sociocultural factors are likely to influence a woman’s beliefs surrounding illness and treatment options; so too might clinician perception of factors affecting transplant candidacy, for example [10]. The complications of CKD are also experienced differently by men and women. The *Chronic Renal Insufficiency Cohort (CRIC)* study reported sex differences in biochemical markers of CKD-mineral bone disease and anaemia [5]. Anaemia is defined by differing haemoglobin thresholds in general medical practice yet current guidelines in CKD follow a one-size-fits-all approach.

## Sex and gender differences in CKD and hypertension: clinical practice

Comparison of health outcomes by sex and/or gender is highly nuanced due to a multitude of confounding factors. The potential reasons for the observed disparities are many-fold and encompass both biological effects (genetic and hormonal factors) and socio-cultural factors on patient-, provider- and system-levels. Clinical outcomes may be difficult to untangle; however, sex and gender disparities relating to the management of CKD and hypertension are measurable, with modifiable targets which may alter clinical outcomes.

### Detection of severity and risk

Among people with CKD, hypertension is almost universal. A documented diagnosis of CKD is associated with better quality cardiovascular disease risk management, including better blood pressure control and prescription of RAS inhibitors [11]. However, women are less likely to receive a diagnosis of CKD than men. In the *Stockholm Creatinine Measurements (SCREAM)* project in over 200,000 Swedish adults with eGFR <60 ml per min per 1.73 m^2^ (55% women): (i) there are not substantial sex differences in serum creatinine measurement; but (ii) in individuals with an eGFR <60 ml per min per 1.73 m^2^, women (3.4%) were less than half as likely as men (6.9%) to have a diagnosis of CKD recorded in their medical records; and (iii) women were less likely to be referred to nephrology services [12]. The problem is not unique to Sweden and similar discrepancies in recognition or awareness of CKD and referral patterns have also been reported in the USA [13, 14].

Albuminuria testing is recommended in the routine management of hypertension [15] as a screening tool for end-organ kidney damage; however, albuminuria is also an important marker of cardiovascular risk [16] and of kidney disease progression [17]. The Kidney Failure Risk Equation (KFRE) is a risk prediction tool which has been validated in multiple international cohorts to quantify the risk of kidney failure, but requires measurement of albuminuria for calculation [17]. In the absence of albuminuria testing, opportunities to identify individuals at the highest risk of kidney failure, and who would benefit from nephrology referral, are missed [18].

A meta-analysis of over 2 million adults with hypertension from the *CKD Prognosis Consortium* estimated the albuminuria screening rate in people with hypertension to be less than 5%, despite over 20% of hypertension cohort participants having prevalent albuminuria (uACR ≥30 mg/g) [19]. Risk of albuminuria in hypertension is comparable to diabetes, yet patients with hypertension are much less likely to be screened: the ratio of undetected to detected albuminuria (uACR ≥30 mg/g) was estimated to be 1.8 in diabetes compared to 19.5 in hypertension [19].

The *SCREAM* project authors particularly highlight sex disparities in albuminuria testing. Albuminuria testing is much less common in women than men: findings which are consistent with studies from the UK [18] and USA [20]. Although overall albuminuria testing has increased over time, the sex gap is stubbornly persistent [12].

**Fig. 2:**
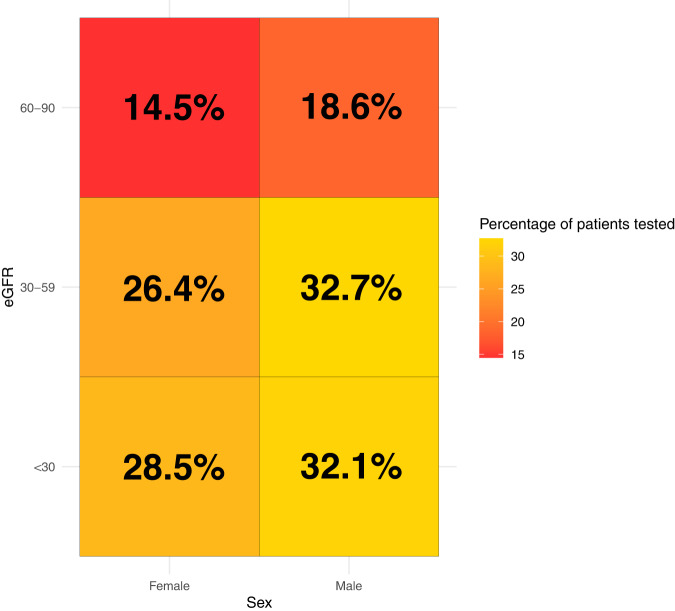
Albuminuria testing in SAIL Databank patients stratified by sex and eGFR. Heatmap colouring reflects the percentage of patients tested in each eGFR and sex category where red represents the lowest percentage tested and yellow represents greater proportions tested; corresponding percentage values are also printed for each category.

Using data from the Secure Anonymised Information Linkage Databank (SAIL), an electronic repository of routinely-collected, de-identified, linkable primary care data for almost 80% of the population of Wales [21], we were able to further explore these patterns in a UK population during 2013–2020. Patients with a diagnosis of hypertension (according to primary care read codes) were identified, stratified by sex and further categorised by estimated glomerular filtration rate (eGFR): <30, 30–59 and 60–90 ml per min per 1.73 m^2^. Patients were included when two blood tests three months or more apart confirmed their kidney function was within a category. Rates of albuminuria testing were recorded within the 12 months following the date of eGFR measurement, as guidelines suggest that albuminuria should be recorded in these patients and within this time frame. The cohort included 427,096 patients of whom those with eGFR between 30 and 59 ml per min per 1.73 m^2^ were most likely to receive testing in primary care (30,561/104,764, 29.2%; Fig. 2). Patients with eGFR <30 ml per min per 1.73 m^2^ are particularly likely to be under the care of nephrology services and secondary care albuminuria testing is not recorded within the primary care-based SAIL. Crucially, sex differences were apparent irrespective of eGFR category in SAIL. Consistent with *SCREAM* reports, men were more likely to undergo albuminuria testing than women (overall occurrence of testing in men: 43 818/196 593, 22.2% versus 41 515/230 503, 18.0% in women, *p* < 0.001; Fig. 2). Low rates of albuminuria testing in people with hypertension—and particularly women with hypertension—may reflect lack of specific guidance in hypertension guidelines on frequency of albuminuria testing; by comparison, diabetes guidelines clearly recommend at least annual albuminuria screening [15, 19].

### Treatment selection

RAS inhibitors are the first-line treatment for many patients with hypertension and are particularly emphasised for individuals with albuminuria. Studies have previously shown that women—due to a combination of genetic and hormonal factors—tend to overexpress angiotensin converting enzyme (ACE) receptors [22, 23], but are less likely to receive RAS inhibitor therapy than men when clinically indicated [12, 24]. This phenomenon must be urgently addressed to ensure equitable access to disease-modifying and life-extending therapies.

Sodium glucose cotransporter-2 (SGLT2) inhibitors have recently been shown, in large randomised placebo-controlled trials, to slow kidney disease progression in CKD down to an eGFR of at least 20 ml per min per 1.73 m^2^ irrespective of blood pressure, yet there is emerging evidence to suggest larger effects in those with higher levels of albuminuria [25]. Albuminuria testing will therefore be key to identifying high-risk patients (such as those with hypertension) who are most likely to benefit.

## Sex and gender differences in CKD and hypertension: clinical research

Randomised controlled trials (“trials”) are the gold standard for evidence generation and key to advancing the management of hypertension and CKD; however, stark sex and gender differences exist. Trials focus on average effects in a selected population and the importance of appropriate evaluation of heterogeneity of treatment effect by sex and gender is increasingly being recognised. In the absence of considering sex and gender heterogeneity, there may be limitations in the external validity and generalisability of trials. The ability to reliably report sex-disaggregated analyses critically requires sex and gender balance in clinical trial participation.

Despite accounting for 55% for the global CKD population, a recent review of 192 CKD trials including almost 150,000 participants found that only 45% of trial participants were women [4]. This figure is even lower at ~33% female representation in recent SGLT2 inhibitor trials in CKD [26]. Underrepresentation of women is particularly apparent in Europe [4]. The problem seems to pertain to recruitment but not retention since trial attrition does not differ substantially in men and women [27]. The reasons for disparate involvement in clinical trials are likely multifactorial and so too must be strategies to overcome them. A recent report of over 600 international National Institutes of Health- and industry-funded trials in cardiovascular disease raises the interesting hypothesis that trials led by female principal investigators are more likely to successfully recruit female participants [28]. A major limitation to this study was the assumption of binary investigator gender based on name; nevertheless, it raises an interesting hypothesis which ought to be further explored. Furthermore, although the proportion of trials led by women is low (less than 20% for the cardiovascular trials analysed), these findings are not out-of-keeping with the demographics of the male-dominated cardiovascular specialties but may differ in other fields.

The underrepresentation of women in trials is a barrier to robust assessment of sex-disaggregated efficacy outcomes. In *Pinho-Gomes* et al’.s review, sex-stratified results were presented for efficacy assessments in only one fifth of trials and none of the 192 trials considered sex differences in safety assessments. Analyses of individual participant-level data in trials of chronic medical conditions show that serious adverse event rates vary by sex across a range of index conditions aggregate [29] due to differential pharmacokinetic and pharmacodynamic effects. Low absolute event rates limit sex subgroup analyses nevertheless trialists must give greater consideration to sex-disaggregated analyses.

## Conclusions

Hypertension is both a cause and consequence of CKD. Clear sex differences exist in CKD identification, risk stratification and management; gender differences are not reported at all. There is a clear paradox between the discrepant burden of CKD in women [4] versus their stagnant underrepresentation in clinical trials and there has been a distinct lack of progress over time [4, 12]. Sex-disaggregated reporting and mixed-methods research will be crucial to improving understanding of the differential impact of hypertension and CKD by sex and gender. Improved understanding must then inform impactful, evidence-based change and influence policy makers, locally and internationally, if health justice is to be achieved.

## Data Availability

Data that support the findings of this study are available from SAIL, subject to successful registration and application process. Further details can be found at https://saildatabank.com.
